# Electroencephalography evidence of functional connectivity modulation and its correlation with bimanual visuomotor learning

**DOI:** 10.1007/s11571-025-10336-9

**Published:** 2025-09-24

**Authors:** Chatrin Phunruangsakao, Chihiro Hosoda, Mitsuhiro Hayashibe

**Affiliations:** 1 Neuro-Robotics Laboratory, Graduate School of Biomedical Engineering, Tohoku University, Sendai, 980-8579 Japan; 2 Department of Cognitive and Behavioral Neuroscience, Institute of Development, Aging and Cancer, Tohoku University, Sendai, 980-8575 Japan; 3 Human Learning and Memory Laboratory, Graduate School of Information Science and Technology, Tohoku University, Sendai, 980-8579 Japan; 4 Department of Robotics, Graduate School of Engineering, Tohoku University, Sendai, 980-8579 Japan

**Keywords:** Visuomotor adaptation, Bimanual movement, Motor learning, Functional connectivity, Electroencephalography

## Abstract

Recent studies have shown that neuroplasticity related to sensorimotor adaptation can occur within short time frames, ranging from minutes to hours. However, it remains unclear whether bimanual training can induce similar effects. Therefore, the objective is to investigate immediate functional brain changes following brief bimanual visuomotor adaptation training. Node and edge-level electroencephalogram functional connectivity analysis and principal component regression were employed to examine changes related to visuomotor tracking task performance. The results revealed significant post-training improvements in bimanual performance, along with decreased node closeness centrality in the non-dominant right frontal and sensorimotor regions within the beta band, as well as in the right frontal, sensorimotor, and occipital regions within the gamma band. Edge-wise analysis indicated reduced beta- and gamma-band connectivity in the right hemisphere, aligning with the node-wise findings. Additionally, theta-band closeness centrality in the frontal, centroparietal, occipital, and temporal regions was positively correlated with bimanual performance, indicating a shift toward more centralized processing as performance increased. Principal component regression further demonstrated its predictive value for bimanual visuomotor performance. This study demonstrates that brief bimanual training elicits immediate functional connectivity changes associated with improved motor performance, particularly reduced right hemisphere beta/gamma connectivity and increased theta centrality. These findings highlight dynamic neural reorganization during bimanual adaptation. However, the interpretation of the results is limited by small sample size, EEG’s low spatial resolution, and bias in functional connectivity estimation. These findings provide insights into adaptation mechanisms that could inform rehabilitation strategies for individuals with motor impairments.

## Introduction

The human sensorimotor control system is a complex network that enables smooth interaction with the environment. A key component of this system is motor adaptation, which continuously refines motor commands based on sensory feedback, allowing for skill improvement over time. This process operates through a loop of prediction, error detection, and correction, ensuring accurate movement execution and gradual enhancement with practice (Leib et al. 2024). One example is visuomotor adaptation, where visual input is integrated with motor actions to adjust to changes in spatial relationships between movement and visual feedback.

Electroencephalography (EEG) is widely used to study neural signals related to visuomotor adaptation due to its high temporal resolution, non-invasiveness, and cost-effectiveness (Reuter et al. 2022). Event-related desynchronization and synchronization (ERDS) refer to decreases and increases, respectively, in the power of specific EEG frequency bands relative to baseline. ERDS has been extensively utilized to study neuroplasticity associated with visuomotor adaptation. For instance, theta (3–7 Hz) activity is more pronounced in complex tasks requiring greater cognitive effort and is particularly elevated during early learning (Reuter et al. 2022). Post-movement beta (13–35 Hz) ERS is sensitive to error size, decreasing when errors are large and increasing when they are small (Desrochers et al. 2020; Alayrangues et al. 2019). Additionally, sensorimotor alpha (7–13 Hz) or mu-band power increases with motor learning and is higher in experts than in novices (Kerick et al. 2004). Pre-movement gamma (35–50 Hz) activity over the prefrontal cortex is also associated with improved re-adaptation (Thürer et al. 2016).

While ERDS analysis provides valuable insights into localized cortical activity, motor learning relies on interactions across multiple brain regions (Phunruangsakao et al. 2024; Virameteekul and Bhidayasiri 2022). Functional connectivity analysis offers a broader perspective by examining how these regions communicate, capturing the integration of information required for learning and adaptation. However, most studies focus on unimanual movements, even though daily activities require coordinated bimanual control. In the context of bimanual visuomotor adaptation, only a few studies have investigated functional connectivity. For instance, Desrochers et al. (2020) found that beta power and coherence within and between hemispheres were initially high but decreased as learning progressed. Similarly, Serrien (2009) reported that beta coherence increased between a left hemisphere–midline network, suggesting a link between higher beta coherence and greater task difficulty.

Neuroplasticity can manifest as either structural or functional changes, with structural changes typically occurring over the long term and being more enduring, while functional changes happen more rapidly. While much of the existing literature on neuroplasticity has focused on changes occurring over extended periods, such as weeks or months, recent studies have demonstrated that both structural and functional neuroplasticity can occur within much shorter time frames, even within an hour or just a few minutes of training. For example, an animal study by Xu et al. (2009) observed signs of structural plasticity within minutes of training. Similarly, Nierhaus et al. (2021) reported modulations in both structural and functional MRI after just one hour of brain-computer interface training. Furthermore, Phang et al. (2021) detected immediate changes in functional connectivity in the parietal-frontocentral regions during post-stroke rehabilitation using music therapy.

Building on the previously discussed concepts, this study aims to investigate how bimanual visuomotor adaptation impacts EEG functional connectivity. The hypothesis is that brief bimanual training induces immediate functional changes, with the degree of changes correlating with improvements in task performance. To test this, two types of visuomotor rotation tasks were applied: a 90-degree counterclockwise rotation and a mirror reversal. EEG connectivity was assessed using corrected imaginary phase-locking values, with analyses performed at the node and edge to examine changes in local node importance and inter-region communication. The relationship between bimanual performance and these connectivity metrics was explored using principal component regression. Furthermore, network activation patterns before and after learning were compared through network-based statistics to identify training-induced changes in functional connectivity.

## Methods

**Fig. 1:**
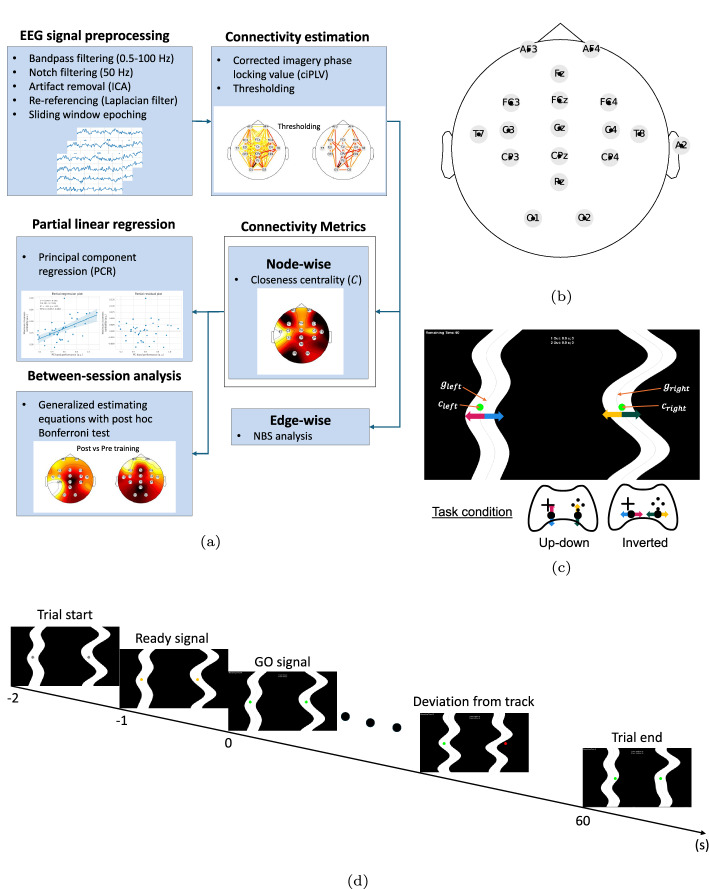
Overview of the experimental design and analysis pipeline. **a** Summary of the analysis pipeline. **b** The 16-channel EEG configuration, with Fz as the ground electrode and A2 as the reference electrode. **c** Diagram illustrating the screen layout during the bimanual visuomotor tracking task; arrow colors represent the direction of the joysticks and cursors, $c$ denotes cursor position, and $g$ denotes track position. **d** Timing of the experimental trial

### Participants

Twenty participants (18 males, 2 females; $M_{Age} = 24.7$, $SD_{Age} = 2.3$) without any known neurological disorders were recruited for the study. All participants had normal or corrected-to-normal vision and were confirmed as right-handed using the Edinburgh Handedness Inventory (Oldfield 1971).

The experimental protocol was approved by the Ethics Committee of the Graduate School of Engineering, Tohoku University (Approval Number: 24A-5). Prior to participation, all individuals provided informed consent following the Declaration of Helsinki. Participants were informed that identifiable data and images may be included in publications, but these would be anonymized or pseudonymized to ensure privacy and confidentiality.

### EEG signal acquisition and preprocessing

The overview of the signal processing and analysis pipeline is summarized in Fig. 1a. EEG signals were acquired and amplified using a 16-channel g.USBamp amplifier (g.tec Medical Engineering GMBH) at a sampling rate of 512 Hz. Wet active Ag/AgCl electrodes were placed at AF3, AF4, FC3, FCz, FC4, C3, Cz, C4, T7, T8, CP3, CPz, CP4, Pz, O1, and O2. The ground electrode was located at Fz, while the reference electrode was attached to the right earlobe (A2), as depicted in Fig. 1b.

An eighth-order Butterworth band-pass filter, with cutoff frequencies set between 0.5 and 100 Hz, was applied to the EEG signals. Furthermore, a fourth-order Butterworth notch filter was employed to reduce power line interference at 50 Hz. Ocular, muscle, and movement artifacts were identified through visual inspection and subsequently removed using independent component analysis. Additionally, the signals were re-referenced using a surface Laplacian filter to enhance localized activity and suppress diffuse signals.

### Experimental design

Participants were positioned approximately 1 m from a display screen, which featured two tracks on the left and right sides for bimanual tracking using joysticks. The goal of the experiment was to assess participants’ adaptability in bimanual visuomotor tracking tasks. Two joystick-to-cursor mappings were used: *Up-down*, in which joystick movements were rotated 90 degrees counterclockwise relative to cursor movements, and *Inverted*, where joystick movements were mirrored relative to cursor movements, as shown in Fig. 1c.

At the start of each trial, the joystick cursors appeared in red, positioned at the center of each track. After a 1-second delay, the cursors changed to yellow, indicating the preparatory phase, and after another second, they turned green, signaling the commencement of the trial (GO signal). Each trial lasted 60 s, during which the tracks moved downward. Participants were required to align the cursors with the tracks. Any deviation or contact with the track boundaries caused the cursors to turn red, prompting participants to realign their tracking, as shown in Fig. 1d.

The experiment consisted of three sessions: pre-training, training, and post-training. In the pre-training and post-training sessions, participants completed four trials–two for each task presented in a random sequence–at the hardest difficulty level. In the training session, six trials were conducted, with three trials for each task in a random sequence. The difficulty gradually increased across these trials, corresponding to easy, moderate, and hard levels. For each difficulty level, the frequency parameter $f$ was drawn from different ranges. Specifically, for the easy level, $f \in \mathcal {U}(0.01, 0.5)$; for the moderate level, $f \in \mathcal {U}(0.01, 0.75)$; and for the hard level, $f \in \mathcal {U}(0.01, 1)$.

The tracks used in the task were generated pseudorandomly by combining 20 sine waves, as described by the equation:

1 $$\begin{aligned} g = \sum _{i=1}^{20} A_i \sin \left( f_i \left( y + \frac{t}{6} \right) + \phi _i \right) \end{aligned}$$

where $g$ is the x-coordinate of the track, $y$ is the y-coordinate, and $t$ represents time. The parameters $A_i \sim \mathcal {U}(1, 10)$ are amplitude parameters drawn from a uniform distribution, and $\phi _i \sim \mathcal {U}(-180, 180)$ are phase parameters also drawn from a uniform distribution. Before starting the experimental trials, participants completed several familiarization trials. A brief break was provided after each trial to minimize fatigue. Behavioral data were sampled at 50 Hz, in line with the monitor’s refresh rate.

Performance was quantified using the Pearson correlation coefficient ($r$) to assess the linear relationship between the cursor positions ($c$) and the track positions ($g$). The formula for $r$ is given by:

2 $$\begin{aligned} r = {\left\{ \begin{array}{ll} \frac{\sum _{i=1}^n (c_i - \bar{c})(g_i - \bar{g})}{\sqrt{\sum _{i=1}^n (c_i - \bar{c})^2} \sqrt{\sum _{i=1}^n (g_i - \bar{g})^2}}, & \text {if } r \ge 0 \\ 0, & \text {if } r < 0 \end{array}\right. } \end{aligned}$$

where $c_i$ and $g_i$ represent the $i$-th data points for the cursor and track positions, respectively, and $\bar{c}$ and $\bar{g}$ are their mean values. $n$ denotes the total number of data points. This constraint ensures the value of $r$ lies between 0 and 1, where values closer to 1 indicate better performance.

The Pearson correlation coefficient was chosen over metrics such as absolute error (AE) or root mean square error (RMSE) because it captures the strength of the linear relationship between cursor and track positions, regardless of scale or offset. This is well-suited for tasks where participants are allowed some deviation and aim to stay within track boundaries rather than precisely follow the centerline. Unlike AE and RMSE, which are sensitive to outliers, $r$ emphasizes co-variation and is less affected by occasional large deviations, making it a more robust indicator of overall trajectory tracking performance.

Performance for each session was calculated by averaging the values of all trials within a session, irrespective of task conditions, for each participant. Moreover, principal component analysis (PCA) was employed to reduce left- and right-hand performance measures into a single dimension, referred to as bimanual performance. This approach is favored over simply averaging the two performances, as PCA maximizes the variance across both hands, accounting for both individual and combined contributions. Importantly, the performance of the left and right hands may differ significantly; thus, averaging the two values could obscure meaningful asymmetries and lead to a loss of critical information regarding hand-specific performance characteristics.

### Functional connectivity analysis

#### Functional connectivity estimation

Prior to functional connectivity estimation, each EEG trial was segmented into epochs using sliding windows. A window length of 4 s and a stride of 2 s were applied, resulting in 120 windows per trial. These windows were subsequently used for functional connectivity analysis.

Brain functional connectivity was estimated using the corrected imaginary phase-locking value (ciPLV) (Bruña et al. 2018), an extended version of the original phase-locking value (PLV) (Lachaux et al. 1999). This method was selected for its robustness against volume conduction and source leakage, its ability to disregard zero-lag connectivity, its sensitivity to non-linear dynamics, and its low computational complexity. The PLV was calculated as follows:

3 $$\begin{aligned} \textrm{PLV} = \left| \mathbb {E} \left[ S_{xy} /\left| S_{xy}\right| \right] \right| \end{aligned}$$

where $\mathbb {E}[\cdot ]$ represents the expected value over windows, and $S_{xy}$ is the cross-spectral density between EEG signals $x$ and $y$.

However, the PLV measure is sensitive to volume conduction and source leakage effects. To address these limitations, the measure was extended to ciPLV, which reduces the influence of these confounding factors by isolating the imaginary component of phase differences and applying normalization. It is formulated as follows:

4 $$\begin{aligned} \textrm{ciPLV} = \frac{\left| \mathbb {E}\left[ \Im \left( {S_{xy}}/{|S_{xy}|}\right) \right] \right| }{\sqrt{1 - \left| \mathbb {E}\left[ \Re \left( {S_{xy}}/{|S_{xy}|}\right) \right] \right| ^2}} \end{aligned}$$

where $\Im (\cdot )$ represents the imaginary part, while $\Re (\cdot )$ represents the real part. The ciPLV was computed for all possible pairs of the 16 electrodes, producing a 16-by-16 connectivity matrix with 120 unique connections, as ciPLV is an undirected measure. The ciPLV values range from 0 to 1, where 0 indicates no phase synchronization and 1 indicates perfect synchronization. The calculations were conducted separately for four EEG frequency bands: theta (3–7 Hz), alpha (7–13 Hz), beta (13–35 Hz), and gamma (35–50 Hz). The resulting values were averaged across sessions, yielding two connectivity matrices (i.e., pre- and post-training sessions) for each participant and frequency band. Function connectivity estimation was carried out using the MNE-Connectivity Python package (Gramfort et al. 2014).

The resulting connectivity matrices may contain weak or spurious connections, which may introduce noise in subsequent analyses; therefore, a percolation analysis was performed to eliminate these connections. The analysis iteratively removes the weakest connections until the graph becomes disconnected, preserving the last connected state. This is analogous to identifying the maximum threshold that removes weak connections while maintaining the full connectivity of the graph.

**Algorithm 1:**
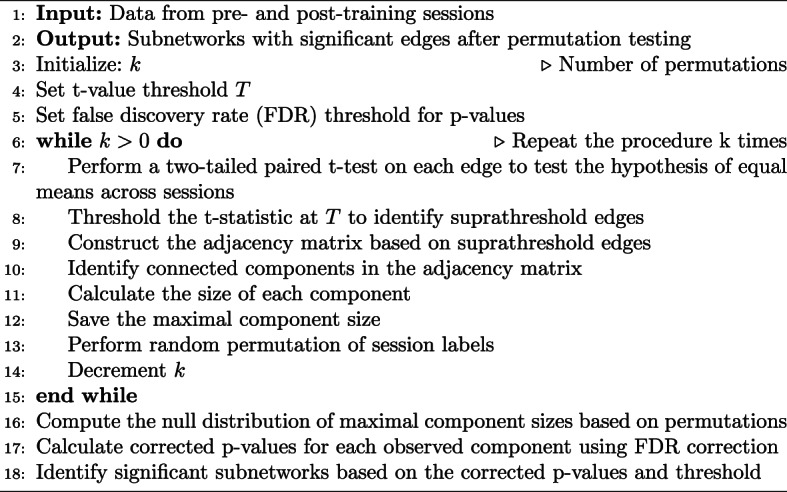
Network-based statistic (NBS) (Adapted from Zalesky et al. (2010))

#### Node-wise analysis

Closeness centrality ($C$) was used to assess the importance or influence of a node within a network. This metric is based on the assumption that a node with a short average path length can interact with many other nodes through only a few intermediate links, indicating its topological centrality (Beauchamp 1965). In other words, nodes with high closeness centrality can quickly reach other nodes in the network, making them crucial for efficient communication or information flow. Closeness centrality is calculated as:

5 $$\begin{aligned} C(i) = \frac{N-1}{\sum _{j \ne i} l(i, j)} \end{aligned}$$

where $N$ is the number of nodes and $l(i, j)$ is the shortest path length between nodes $i$ and $j$. Note that the path length here is defined as the reciprocal of the weight, which is calculated by ciPLV, because higher connectivity (represented by stronger connections) corresponds to shorter path lengths, reflecting more efficient communication between nodes.

#### Edge-wise analysis

The network-based statistics (NBS) toolbox (Zalesky et al. 2010) was used to detect subnetworks with edges showing significant differences between sessions while controlling for the family-wise error rate. The null hypothesis assumes that the edge weights or connectivity strengths have equal means across the two sessions. The key steps of the NBS procedure are outlined in Algorithm 1. Changes in network components were assessed using a t-value threshold ($T$) of 2.75. Permutation testing ($k = 100,000$) was subsequently applied to calculate the *p*-value. Statistical significance was determined with a threshold of $\alpha = 0.05$.

#### Correlation analysis and linear regression model

For each frequency band, the goal was to determine how well the node metric predicted behavioral performance. As the metrics may introduce multicollinearity in linear regression, principal component regression (PCR) was applied. Initially, performance data from both hands were reduced to a single component using principal component analysis (PCA). As a feature selection step, Spearman correlations between the bimanual performance and node closeness centrality were computed. However, if participants showed improved performance between sessions, this training effect may be associated with changes in connectivity. Hence, correlations between performance and connectivity could be confounded by these session-related or participant-specific effects. To address this potential confounding, partial correlations were computed while controlling for both session and participant. If the partial Spearman correlations, after Bonferroni correction with a significance threshold of $\alpha = 0.05$, were found to be significant, partial ordinary least squares regression was then conducted using the following formula:

6 $$\begin{aligned} y' = \beta _0 x' + \beta _1 \end{aligned}$$

where $y'$ represents the residual of the dependent variable $y$ (bimanual performance) after removing the effect of the control variables. Similarly, $x'$ is the residual of the predictor variable after accounting for the influence of the control variable. $\beta _0$ and $\beta _1$ are the slope and intercept of the model, respectively. The model was trained and evaluated using leave-one-out cross-validation to evaluate the predictability.

Connectivity strength was not included in the analysis, as the percolation process led to a high number of zero connections, which could diminish the statistical power of both correlation and regression analyses.

### Statistical analysis

Statistical analyses were conducted using R (version 4.4.3), focusing on group-level effects. Due to the non-normality and skew of the data, a generalized estimating equations (GEE) approach with a gamma log link function was employed to evaluate differences. This was implemented using the geepack package (Højsgaard et al. 2006). GEE accounts for within-subject correlations by incorporating a dependence structure, with an exchangeable correlation structure assumed in this analysis.

To ensure adequate statistical power for detecting a within-subject effect, a sample size estimation was performed using the lmmpower function from the longpower package (Iddi and Donohue 2022; Diggle 2002). Assuming an expected slope of .6, a residual standard deviation of .5, an intra-subject correlation of .6, and two repeated measurements per subject, the analysis indicated that a sample size of N = 18 would be required to achieve.8 power at a significance level of .05.

For the node-wise analysis, closeness centrality was modeled as a function of session, node, and their interaction. Participants were treated as the clustering variable to account for repeated measures at the group level.

To further interpret significant effects, estimated marginal means (EMMs) were computed using the emmeans package (Lenth 2025). Pairwise comparisons were performed for the session $\times$ node interaction (node-wise), with a Bonferroni correction applied for multiple comparisons. Statistical significance was set at $\alpha <.05$.

A similar GEE model was applied to the behavioral analysis, examining the effects of session, hand (left vs. right), and their interaction on behavioral performance. Additionally, edge-wise statistical analysis was performed using NBS as described in Sect. 2.4.

## Results

**Table 1:** Results of the GEE analysis examining the effects of session, hand, and their interaction on behavioral performance

Variable	df	$\chi ^2$	$p$-value
Session	1	36.238	$\mathbf {< .001}$
Hand	1	2.108	.146
Session: Hand	1	2.070	.150

Only the main effect of session was significant. Bold *p*-values indicate statistically significant effects ($p <.05$)

**Fig. 2:**
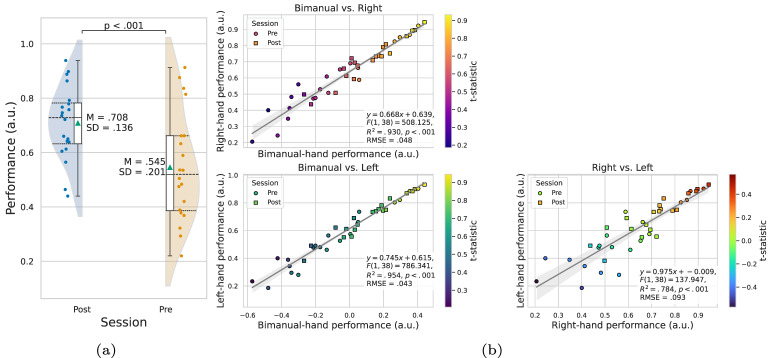
Summary of behavioral performance. **a** Performance comparison between sessions, demonstrating a statistically significant difference between sessions, with no significant differences between hands or interaction effects. Green triangles indicate mean values. **b** Linear regression analysis of bimanual, left-hand, and right-hand performance. Bimanual performance significantly predicts both left- and right-hand performance, while right-hand performance also significantly predicts left-hand performance. Circles and squares represent pre-training and post-training data for each participant, respectively. The gray line indicates the fitted linear regression, and the shaded region shows the 95% confidence interval

**Fig. 3:**
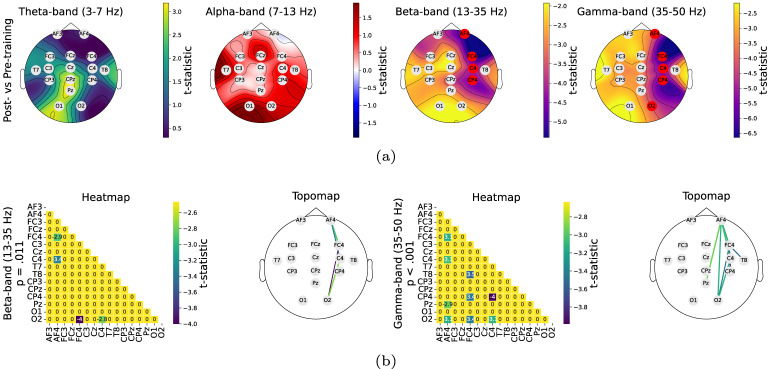
Between-session comparisons. **a** The node-wise analysis revealed significant decreases in node closeness centrality in the beta and gamma bands, particularly within the right hemisphere; nodes with significant changes are highlighted in red. **b** The edge-wise analysis revealed a significant decline in functional connectivity in the beta and gamma bands, with most reductions occurring in the non-dominant right hemisphere

**Table 2:** Results of the GEE analysis examining the effects of session, node, and their interaction on node closeness centrality across different frequency bands

Band	Theta			Alpha			Beta			Gamma		
Variable	df	χ_2_	*p*-value	df	χ_2_	*p*-value	df	χ_2_	*p*-value	df	χ_2_	*p*-value
Session	1	4.388	**.036**	1	0.971	.324	1	17.531	**< .001**	1	17.204	**< .001**
Node	15	383.144	**< .001**	15	1244.845	**< .001**	15	1375.327	**< .001**	15	161.968	**< .001**
Session: node	15	47.967	**< .001**	15	84.751	**< .001**	15	48.511	**< .001**	15	116.033	**< .001**

Significant interaction effects were observed in all frequency bands. Bold p-values indicate statistically significant effects ($p <.05$)

**Table 3:** Node closeness centrality shows high variance inflation factor (VIF), suggesting the presence of collinearity

Node	AF4	T7	CP3	CPz	Pz	O1	O2
VIF	42.536	19.309	20.927	94.976	150.695	67.171	57.122

**Fig. 4:**
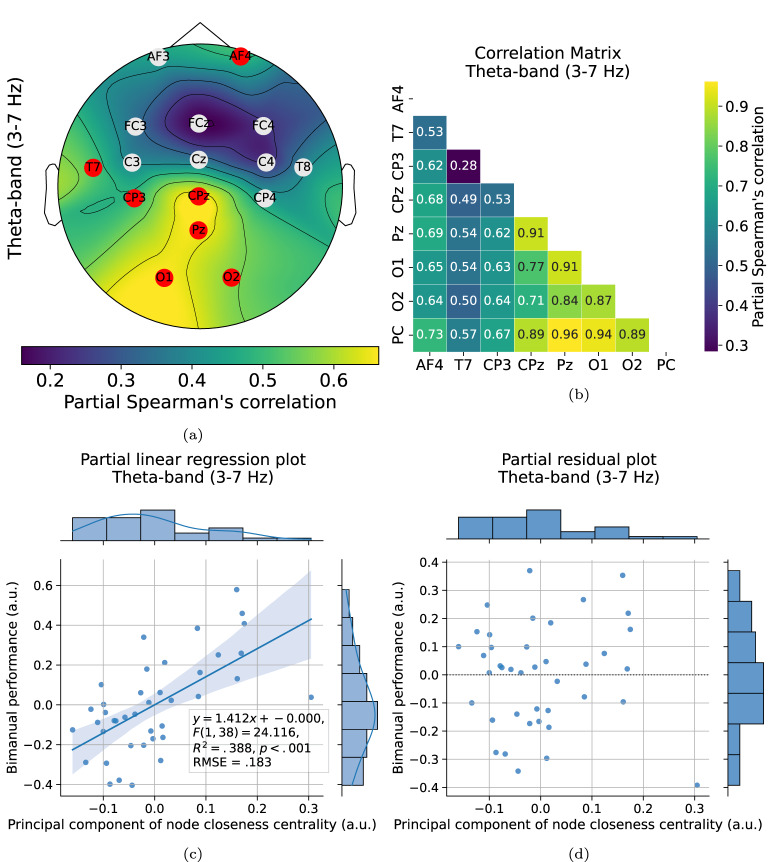
Node-wise correlation and regression analysis on whole dataset. **a** Topographic map showing Spearman partial correlation, with significant correlations (red nodes) primarily observed in frontal, centroparietal, parietal, and occipital regions. **b** Pairwise correlation matrix of significant nodes and the principal component of node closeness centrality (PC). **c** Partial linear regression plot and **d** Residual plot demonstrating that the principal component of closeness centrality in the theta band significantly predicted bimanual performance. Circles represent data from each session and participant, while the blue line denotes the fitted linear regression, with the shaded area representing the 95% confidence interval

**Fig. 5:**
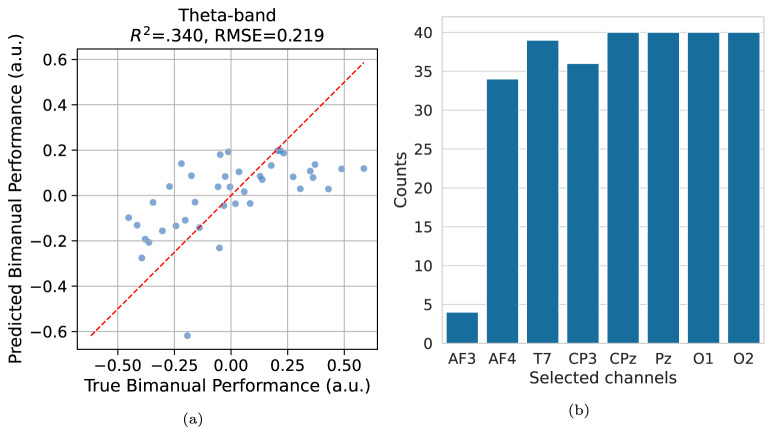
Leave-one-out cross-validation of principal component regression analysis. **a** Prediction of bimanual performance from the theta-band node closeness centrality using principal component regression. The red line indicates identity. **b** Frequency of selected EEG channels across cross-validation folds

### Brief bimanual learning induces significant improvements in performance

The results from the GEE analysis on behavioral performance are summarized in Table 1. A significant main effect of session was observed ($\chi ^2(1) = 36.238$, $p <.001$), with the post-training session ($M =.708$, $SD =.136$) showing higher performance compared to the pre-training session ($M =.545$, $SD =.201$), as illustrated in Fig. 2a

### Dimensionality reduction of bimanual performance

Figure 2b presents the linear regression relationship between left- and right-hand performance and their corresponding principal component. For simplicity, the principal component of both hands is referred to as bimanual performance. The results show that the principal component explains 94.3% of the variance, effectively capturing most of the variability in bimanual performance. Linear regression analysis was conducted to assess the predictive ability of bimanual performance for left- and right-hand performance. Significant regression was observed for both the left hand ($F(1, 38) = 786.341$, $R^2 =.954$, $p <.001$) and the right hand ($F(1, 38) = 508.125$, $R^2 =.930$, $p <.001$). Furthermore, right-hand performance was found to significantly predict left-hand performance ($F(1, 38) = 137.947$, $R^2 =.784$, $p <.001$). Due to these findings, bimanual performance derived from PCA was used for subsequent analysis.

### Immediate changes observed in the beta and gamma bands after brief bimanual learning

The node-wise analysis investigated changes in node closeness centrality, with higher values indicating a greater reliance on specific nodes for information transfer. To assess the group-level effects of session and node on closeness centrality, a GEE approach was applied. The results, summarized in Table 2, revealed a significant main effect of session in the theta ($\chi ^2(1) = 4.388$, $p =.036$), beta ($\chi ^2(1) = 17.531$, $p <.001$), and gamma bands ($\chi ^2(1) = 17.204$, $p <.001$). The main effect of node was significant across all frequency bands: theta ($\chi ^2(15) = 383.144$, $p <.001$), alpha ($\chi ^2(15) = 1244.845$, $p <.001$), beta ($\chi ^2(15) = 1375.327$, $p <.001$), and gamma ($\chi ^2(15) = 161.968$, $p <.001$). Additionally, a significant interaction between session and node was found in the theta ($\chi ^2(15) = 47.967$, $p <.001$), alpha ($\chi ^2(15) = 84.751$, $p <.001$), beta ($\chi ^2(15) = 48.511$, $p <.001$), and gamma bands ($\chi ^2(15) = 116.033$, $p <.001$).

A post hoc Bonferroni test was performed to assess the significant differences in node closeness centrality between the post-training and pre-training sessions. The results showed no significant effects in the theta and alpha bands. However, significant differences were found in the beta and gamma bands. In the beta band, notable decreases in closeness centrality were observed in the right frontal (AF4: $t(608) = -5.408$, $p <.001$, $\beta = -0.276$, $SE = 0.051$) and sensorimotor regions (FC4: $t(608) = -5.248$, $p <.001$, $\beta = -0.252$, $SE = 0.048$; C4: $t(608) = -4.202$, $p =.015$, $\beta = -0.225$, $SE = 0.053$; CP4: $t(608) = -4.551$, $p =.003$, $\beta = -0.258$, $SE = 0.057$) after the brief bimanual training. In the gamma band, significant reductions in closeness centrality were found in the right frontal region (AF4: $t(608) = -4.054$, $p =.028$, $\beta = -0.360$, $SE = 0.089$), sensorimotor regions (FC4: $t(608) = -6.644$, $p <.001$, $\beta = -0.417$, $SE = 0.063$; C4: $t(608) = -5.432$, $p <.001$, $\beta = -0.389$, $SE = 0.072$; CP4: $t(608) = -5.864$, $p <.001$, $\beta = -0.404$, $SE = 0.069$), and the occipital region (O2: $t(608) = -4.094$, $p =.024$, $\beta = -0.305$, $SE = 0.075$) following the brief bimanual training.

The edge-wise analysis investigated the differences in connectivity strength between node pairs using network-based statistics. As illustrated in Fig. 3b, two distinct networks were identified in both the beta and gamma bands. The significant network in the beta band ($p =.011$) consisted of four connections within the right hemisphere, specifically involving the right frontal (AF4), frontocentral (FC4), central (C4), and occipital (O2) nodes, reflecting a consistent trend observed in the node-wise analysis. A similar pattern was observed in the gamma band ($p <.001$), though the network expanded to include nine connections and incorporated additional nodes in the parietal (Pz), right temporal (T8), and right centroparietal (CP4) regions.

The overall trend observed across the node- and edge-wise analyses indicates a consistent decrease in functional connectivity within the beta and gamma bands following brief bimanual training. Reductions in node closeness centrality were observed in the beta and gamma bands. The edge-wise analysis revealed significant reductions in connectivity in regions similar to those identified in the node-wise analysis within the right hemisphere in both the beta and gamma bands. Collectively, these results suggest that brief bimanual training led to a reduction in connectivity strength in both the beta and gamma bands and a decreased reliance on individual nodes for information transfer, particularly within the right hemisphere.

### Bimanual performance improves with increased node reliance in the theta band

The partial Spearman’s correlation analysis, shown in Fig. 4a, identified a significant correlations between node closeness centrality and bimanual performance in the theta band across eight nodes: AF4 ($\rho =.485$, $p =.033$, $95\%CI = [.20,.70]$), T7 ($\rho =.518$, $p =.014$, $95\%CI = [.24,.72]$), CP3 ($\rho =.501$, $p =.021$, $95\%CI = [.22,.71]$), CPz ($\rho =.663$, $p <.001$, $95\%CI = [.44,.81]$), Pz ($\rho =.659$, $p <.001$, $95\%CI = [.43,.81]$), O1 ($\rho =.661$, $p <.001$, $95\%CI = [.43,.81]$), and O2 ($\rho =.571$, $p =.003$, $95\%CI = [.31,.75]$). Notably, all correlations were positive, indicating that higher closeness centrality in these nodes is associated with improved bimanual task performance.

Next, the objective was to assess whether node closeness centrality could significantly predict bimanual performance. However, as illustrated in Fig. 4b, the nodes with significant correlations also demonstrated strong intercorrelations. Table 3 reports high variance inflation factors for all nodes, indicating potential multicollinearity in the linear regression model, which may undermine its predictive performance. To mitigate this issue, PCR was employed. The significant nodes were reduced to a single dimension via PCA, which accounted for 80.8% of the variance, deemed sufficient for the analysis. Partial linear regression analysis demonstrated that the principal component of node closeness centrality significantly predicted bimanual performance ($F(1, 38) = 24.116$, $R^2 =.383$, $p <.001$). The partial linear regression and residual plots are presented in Figs. 4c, d, respectively. The predictive validity of regression model, shown in Fig. 5a, was further confirmed using leave-one-out cross-validation, yielding satisfactory predictability ($R^2 =.340$, $RMSE = 0.219$). Furthermore, Fig. 5b displays the frequency of selected channels for regression, which aligns with the pattern observed previously. This analysis shows a robust linear relationship between theta-band node centrality, particularly in the frontal, centroparietal, parietal, and occipital areas, and bimanual performance.

## Discussion

This study employed two levels of analysis to investigate the effects of brief bimanual visuomotor training on brain functional connectivity during task execution. The primary goals were (1) to assess group-level changes in functional connectivity between pre- and post-training sessions, and (2) to explore the relationship between bimanual performance and node metrics. The results revealed a significant improvement in bimanual performance following the training. Specifically, reductions in node closeness centrality were observed in the non-dominant right frontal and sensorimotor regions within the beta band (13–35 Hz), as well as in right hemisphere regions, including frontal, sensorimotor, and occipital areas, in the gamma band (35-50 Hz). Edge-wise analysis similarly showed decreased connectivity in the beta and gamma bands, particularly in the right hemisphere, aligning with the node-wise findings. These findings suggest that brief bimanual training leads to reduced beta- and gamma-band connectivity and a diminished reliance on individual nodes for information processing, especially in the right hemisphere. Furthermore, closeness centrality in the frontal, centroparietal, parietal, temporal, and occipital regions within the theta band (3-7 Hz) was positively correlated with bimanual performance and significantly predicted it, indicating that better performance is associated with a more centralized, node-dependent processing strategy in the theta band.

Motor skill learning occurs across multiple timescales, characterized by rapid initial performance gains followed by slower, asymptotic improvements as the skill becomes increasingly refined, ingrained, and automatic (Dayan and Cohen 2011). The neural circuits and mechanisms involved in fast and slow learning differ (Kim et al. 2015). Fast learning is driven by short-term functional changes. Whereas, slow learning leads to long-lasting structural changes, such as increased axon growth, synapse formation, dendritic density, and alterations in grey matter (Dayan and Cohen 2011; Murray and Escola 2020). For example, musicians demonstrate a larger corpus callosum (Schlaug et al. 1995), while London taxi drivers exhibit an enlarged hippocampus (Maguire et al. 2006). However, recent studies have demonstrated that the adult brain is capable of both functional and structural changes following brief training or stimulation, reflecting early performance gains. For instance, Nierhaus et al. (2021) showed that after one hour of brain-computer interface training, structural changes occurred in regions such as the sensorimotor, medial parietal, and occipital areas, along with functional changes in the inferior frontal gyrus, precuneus, occipital, and medial sensorimotor regions. Similarly, Phang et al. (2021) observed immediate functional connectivity changes in the parietal-frontocentral regions during post-stroke rehabilitation using music therapy. Moreover, astrocytic processes surrounding synapses have been found to become more active within five minutes of stimulation-induced long-term potentiation, suggesting a rapid response to synaptic activity that may play a role in synaptic strengthening (Perez-Alvarez et al. 2014). Although brain structural changes were not investigated in the present study, the immediate changes in brain functional connectivity are interpreted as being induced by the brief bimanual training, which may reflect the early phase of motor learning.

The theory of motor lateralization suggests that each hemisphere specializes in distinct motor control mechanisms. The left hemisphere primarily employs predictive control, translating target, arm position, and task dynamics into sequential commands, while the right hemisphere utilizes impedance control, adjusting movement based on resistance and countering unexpected perturbations (Mutha et al. 2011, 2012). Intermanual transfer enables training one hand to enhance the other’s performance, though each hemisphere selectively acquires skills aligned with its specialization (Mutha et al. 2012; Grafton et al. 2002). This independent yet integrated processing supports effective bimanual coordination and enhances cognitive performance (Güntürkün et al. 2020; van der Knaap and van der Ham 2011; Takeuchi et al. 2012; Tantawanich et al. 2025). This transfer mechanism may explain the absence of interaction effects between hands across sessions.

Event-related desynchronization and synchronization (ERDS) refer to the task-related decrease and increase, respectively, in the amplitude power of specific EEG frequency bands relative to baseline. ERDS has been extensively studied in the context of visuomotor adaptation and is thought to reflect the recruitment of brain regions involved in task execution. Similarly, measures such as node closeness centrality and functional connectivity are considered indicators of network efficiency and functional integration. Reductions in these measures may suggest a shift toward more localized processing in task-relevant areas and diminished communication among task-irrelevant regions.

Alterations in beta-band ERDS and functional connectivity are known to play distinct roles in attention and sensorimotor control (Reuter et al. 2022). Stronger interregional beta-band synchronization is typically associated with increased task complexity. However, with repeated exposure to a perturbation, beta-band connectivity tends to decrease, reflecting improved task proficiency (van Wijk et al. 2012). In this context, a reduction in node closeness centrality may similarly indicate enhanced proficiency, as the system becomes less reliant on specific nodes for information processing and integration.

The asymmetric bimanual tracking task used in this study likely engaged higher-order control mechanisms, increasing the demand on the right hemisphere for sensorimotor processing and on the left hemisphere for spatiotemporal integration. Serrien (2009) demonstrated that neural responses to visuomotor discordance during bimanual mirror drawing occur bilaterally in the beta band, driven by motor and spatial attention mechanisms. Chung et al. (2017) further showed that beta-band connectivity in the sensorimotor and parietal cortices is essential for online motor adaptation. Likewise, Ding et al. (2023) found that beta-band connectivity among the bilateral motor cortex, somatosensory areas, and the inferior frontal cortex correlates with better performance in motor inhibition tasks. Despite these bilateral contributions, bimanual motor behavior is predominantly governed by the left hemisphere, which may transfer control to the right hemisphere via intermanual transfer (Mutha et al. 2011; Haaland et al. 2004; Takeuchi et al. 2012). Accordingly, the observed decrease in node centrality in the right hemisphere–including frontal and sensorimotor regions–may reflect reduced reliance on right-hemisphere mechanisms for successful adaptation. Meanwhile, the lack of changes in the left hemisphere may indicate its continued involvement in sensory integration necessary for adaptation.

Gamma-band ERDS has also been linked to motor control. While gamma oscillations are widely studied in vision and attention research for their role in visual information integration, they are also believed to support cortical communication by synchronizing sensorimotor activity across brain regions (Ulloa 2022). Motor training has been associated with decreased gamma-band connectivity between frontal and motor-related regions, as well as between the frontal lobe and right hemisphere (Gentili et al. 2015), consistent with the edge-wise findings of the present study. This reduction is thought to reflect a shift from feedback to feedforward control, whereby improved performance and increased task automaticity reduce the neural demands of sensorimotor integration and movement correction. Similarly, the observed decrease in gamma-band connectivity between premotor and visual regions may indicate reduced reliance on visual feedback and improved coordination of movement timing. This aligns with findings by Burgos et al. (2018), who reported more pronounced reductions in connectivity in a combined practice group than in a group performing bimanual adaptation alone.

Collectively, the beta- and gamma-band results suggest that reductions in node closeness centrality and functional connectivity may serve as markers of enhanced network efficiency and integration. These changes likely reflect a transition toward more localized processing within task-relevant regions and reduced interregional communication in task-irrelevant areas following training.

The frontoparietal network encompasses regions such as the dorsal frontal cortex, dorsolateral prefrontal cortex, inferior parietal lobe, intraparietal sulcus, lateral parietal cortex, and medial cingulate cortex. This network is essential for coordinating executive control in a rapid, accurate, and flexible manner, active during both resting and task states (Marek and Dosenbach 2018). A study by Hacker et al. (2017) revealed that the frontoparietal network strongly couples with the blood-oxygen-level-dependent signal in the theta band during electrocorticographic recordings. Therefore, the significant correlation observed between bimanual performance and functional changes in the frontal, centroparietal, and parietal regions within the theta band may indicate recruitment of the frontoparietal network. In line with this, a significant correlation between frontoparietal network integration and overall cognitive ability suggests that stronger functional connectivity within this network is associated with enhanced cognitive performance (Sheffield et al. 2015). Additionally, synchronization within the theta and alpha bands across regions of the frontoparietal network is crucial for cognitive control, particularly in tasks that require switching between different rule sets and visuomotor manipulations (Haegens et al. 2010; Klimesch et al. 2007; Kawasaki et al. 2014). In motor control, the frontoparietal network is also responsible for initiating control and providing flexibility by adjusting control in response to feedback. The present study involved two distinct bimanual visuomotor rotation tasks, requiring participants to constantly adapt to perturbed visual feedback and switch between tasks, which may help explain the observed findings.

In addition to the frontoparietal network, the results also identified significant connectivity correlations in the temporal and occipital regions. The frontoparietal network is known to interact with other brain areas to regulate cognitive functions. Specifically, Reinhart and Nguyen (2019) found that working memory involves local and long-range neural circuits, driven by theta–gamma phase-amplitude coupling in the temporal cortex and theta phase synchronization across the frontotemporal cortex. Furthermore, theta-band oscillations in the occipital region are associated with visuospatial attention, while alpha-band oscillations in the occipital cortex support top-down modulation of perception and working memory (Marek and Dosenbach 2018; Cartier et al. 2012). Overall, bimanual visuomotor learning appears to enhance the interaction of the frontoparietal network with other brain regions, facilitating the regulation of working memory, visuospatial attention, and goal-directed behaviors.

Although this study presents promising results, three main limitations should be addressed in future research. First, the participant group was restricted to right-handed males within a narrow age range, limiting the generalizability of the findings, as motor learning processes are influenced by age, sex, and handedness (Takeuchi et al. 2012; Tantawanich et al. 2025). For instance, Lissek et al. (2007) found that during a self-paced finger-tapping task, females showed greater cortical activation, whereas males exhibited stronger striatal activity. Moreover, while women have a proportionally larger corpus callosum, men exhibit greater microstructural connectivity, which may influence interhemispheric interaction necessary for bimanual movements. Several MRI studies have also shown that aging leads to corpus callosum atrophy, deteriorating the interhemispheric interaction (Takeuchi et al. 2012; Takeda et al. 2003). Additionally, right-handed individuals tend to display more bilateral activation patterns than left-handed individuals during motor tasks (Lajtos et al. 2023). These issues emphasize the importance of diverse sampling. Second, the use of a 16-channel low-density EEG system limited the spatial resolution of neural recordings. EEG is inherently susceptible to volume conduction, which can obscure the precise localization of neural sources. Moreover, the limited number of electrodes precluded source-level analysis. While this setup provided adequate coverage of key sensorimotor regions, it does impose constraints on spatial specificity. Future studies should consider employing high-density EEG systems or complementary neuroimaging modalities, such as fMRI, to improve spatial resolution and better elucidate the underlying neural mechanisms. Nonetheless, Justesen et al. (2019) suggested that the duration of EEG recording has a greater impact on diagnostic yield than increased electrode density, indicating that the setup used in this study may have been sufficient for the study’s objectives. Third, although ciPLV effectively mitigates spurious connectivity caused by volume conduction, it remains a bivariate and non-directional measure. Therefore, it does not capture interactions among multiple brain regions, lacks information on directionality, and is limited to frequency-domain analysis. Future research may benefit from applying multivariate and directional connectivity measures–such as multivariate transfer entropy (Ursino et al. 2020) or partial directed coherence (Baccalá and Sameshima 2001)–to more accurately characterize the complexity of brain network dynamics.

## Conclusion

The current study demonstrates that brief bimanual visuomotor training results in significant functional connectivity changes, as evidenced by reductions in node closeness centrality and connectivity in non-dominant right hemisphere regions. Notably, the observed correlation between theta-band closeness centrality and performance suggests that training facilitates a reorganization of neural networks towards more centralized processing as performance increases. These findings highlight the brain’s capacity for rapid neuroplastic adaptation following brief bimanual training, providing a foundation for future research aimed at understanding and enhancing motor rehabilitation strategies for individuals with motor deficits. However, the results should be interpreted with caution due to the small sample size, EEG’s limited spatial resolution, and the inherent bias in functional connectivity estimation.

## Data Availability

The data that support the findings of this study are available from the corresponding author, upon reasonable request.
